# Role of Toll-Like Receptor 2 in Regulation of T-Helper Immune Response in Chronic Obstructive Pulmonary Disease

**DOI:** 10.1155/2021/5596095

**Published:** 2021-08-13

**Authors:** Karolina A. Sidletskaya, Tatyana I. Vitkina, Yulia K. Denisenko, Elena E. Mineeva

**Affiliations:** Vladivostok Branch of Federal State Budgetary Science Institution “Far Eastern Scientific Center of Physiology and Pathology of Respiration”–Institute of Medical Climatology and Rehabilitative Treatment, Russian Street 73-g, Vladivostok 690105, Russia

## Abstract

**Objective:**

According to modern views, the differences in the clinical course of chronic obstructive pulmonary disease (COPD) are associated with certain types of T-helper (Th) immune response. Recent data have shown that toll-like receptor 2 (TLR2) is involved in the development of Th immune response. However, TLR2-mediated regulation of Th subpopulation balance in COPD needs to be elucidated. The aim of our work is to determine the mechanisms of TLR2-mediated regulation of Th immune response in COPD of varying severity.

**Methods:**

The study included 323 smokers/ex-smokers with stable COPD (GOLD I, GOLD II, and GOLD III) and 97 healthy nonsmokers (control group). Serum levels of Th1 (TNF-*α* and IFN-*γ*), Th2 (IL-4), Th17 (IL-6 and IL-17A), Treg (IL-10) cytokines, and the percentage of peripheral blood Th cells expressing TLR2 (CD4^+^CD282^+^) were assessed by flow cytometry. Serum concentrations of IL-21 (Th17) and TGF-*β*1 (Treg) were measured using the ELISA method. The predominant Th cytokine profile in serum was determined by calculating the ratios between levels of Th1 and Th17 cytokines. Spearman's correlation test was performed.

**Results:**

Patients with COPD GOLD II and III with Th1 and Th17 cytokine profiles exhibited an increase in the percentage of CD4^+^CD282^+^ cells compared to the control group. In COPD GOLD I–III, positive correlations between CD4^+^CD282^+^ cell frequency and Th17 cytokine levels (IL-6, IL-17A, and IL-21) were found. In COPD GOLD I, IL-10 concentration was negatively correlated with the percentages of studied cells; in COPD GOLD II, a positive correlation between these parameters was noted.

**Conclusions:**

Enhanced TLR2 expression on CD4^+^ cells shifts cytokine profile toward Th17 phenotype that plays a crucial role in COPD progression. The level of TLR2 expression on peripheral blood CD4^+^ cells may be considered as a biomarker for diagnosing and predicting the progression of COPD.

## 1. Introduction

Nowadays, chronic obstructive pulmonary disease (COPD) is one of the most common chronic inflammatory diseases of the respiratory system [1, 2]. According to modern views, there are several pathophysiological COPD phenotypes with different clinical courses of the disease [3–5].

Recent research data indicate that the classification of COPD into pathophysiological phenotypes is based on a shift in the balance of subpopulations of T-helper (Th or CD4^+^) cells, such as Th type 1 cells (Th1), Th2, Th17, and T-regulatory cells (Treg) [6]. Predominant activation of certain Th subpopulations, which depend on the cytokine microenvironment, determines adaptive immune response involving various mediators and effector cells [7, 8]. The Th1 immune response is characterized by the enhanced production of pro-inflammatory cytokines: tumor necrosis factor-*α* (TNF-*α*) and interferon-*γ* (IFN-*γ*) that trigger the macrophage-mediated inflammatory response. The cytokine IFN-*γ* is also involved in the differentiation of Th1 cells through a feedback loop [7, 9]. Th2-lymphocytes mainly produce IL-4 that is essential for their differentiation and promotes the development of immune response involving mast cells and eosinophils [7, 8]. The development of Th17 immune response is accompanied by an increase in the concentration of IL-17 that is responsible for recruiting neutrophils in the small airways. The essential factors for Th17 cell differentiation are IL-6 and IL-21 [10, 11]. The main function of Treg is to inhibit the activity of effector cells by secreting anti-inflammatory cytokine IL-10 and transforming growth factor-*β* (TGF-*β*) [12, 13].

According to the literature, many reactions of cellular immunity, including the cytokine response, are mediated by toll-like receptors (TLRs). The activation of these receptors occurs due to the binding of molecular structures of pathogens (pathogen-associated molecular patterns, PAMPs) and tissue damage products (damage-associated molecular patterns, DAMPs). In humans, 10 TLRs have been identified. They differ in ligands, signaling pathways, and functions. TLRs may be located on the endosomal membrane (TLR3, TLR7, TLR8, and TLR9) or the cytoplasmic membrane of the cell (TLR1, TLR2, TLR4, TLR5, TLR6, and TLR10) [14].

A number of publications have reported the participation of endosomal TLRs recognizing viruses, such as TLR3, TLR7, and TLR9, in the pathogenesis of COPD [15–20]. However, numerous studies indicate the considerable contribution of membrane TLR2 and TLR4 activated by bacterial PAMPs to the development of the inflammatory process in COPD [21–25]. Altered expression of these TLRs in the tissues of the respiratory tract has been found both in patients with COPD and experimental animals [21, 26–29]. Genetic studies have shown that polymorphism of the TLR2 and TLR4 genes is associated with the risk of developing and progressing COPD [30–32].

As for the role of TLRs in the regulation of Th immune response, there is currently evidence of the direct involvement of TLR2 in this process in various pathologies [33–38], which makes this receptor an interesting object for research. Nevertheless, TLR2-related mechanisms determining the development of the Th immunoregulatory pathway in COPD are still poorly understood. Taking into account the important role of T-helper immune pathways in COPD progression, the determination of TLR2-mediated mechanisms regulating the balance of Th subpopulations in COPD offers significant potential to improve the methods for diagnosing COPD, enable new personalized approaches to treatment, and enhance the quality of prediction of disease course.

The aim of our work is to determine the mechanisms of TLR2-mediated regulation of Th immune response in COPD of varying severity.

## 2. Materials and Methods

### 2.1. Subjects

The study was approved by the Ethics Committee of Vladivostok Branch of Federal State Budgetary Science Institution “Far Eastern Scientific Center of Physiology and Pathology of Respiration”–Institute of Medical Climatology and Rehabilitative Treatment and carried out in accordance with the standards of the Declaration of Helsinki (revised 2013). All subjects gave written informed consent prior to the survey. The study involved 323 smokers/ex-smokers with stable COPD, including 107 patients with COPD GOLD I (mean age 55.9 ± 2.5), 132 patients with COPD GOLD II (mean age 56.8 ± 4.2), and 84 patients with COPD GOLD III (mean age 61.3 ± 2.4) [2]. COPD was diagnosed based on patient medical history, physical examination, peak flowmetry, spirometry with bronchodilator test (MasterScreen Body plethysmograph, Germany), chest X-ray, laboratory tests, mMRC (modified British Medical Research Council) test, and CAT (COPD Assessment Test). The diagnosis was made in accordance with the Global Strategy for Diagnosis, Management, and Prevention of COPD [2]. Clinical characteristics of the examined groups are presented in Table 1. The exclusion criteria were COPD exacerbations during the last 2 months, acute pathological conditions, exacerbation of chronic diseases, and occupational hazards associated with inhalation of toxic substances. All COPD patients were treated with long-acting m-cholinolytics, and patients with COPD GOLD III were treated with long-acting m-cholinolytics combined with a fixed combination of long-acting *β*2-agonists and inhaled glucocorticoids [2]. The control group consisted of 97 practically healthy nonsmokers with normal respiratory function (mean age 51.3 ± 2.5 years).

### 2.2. Determination of Predominant Serum Th Cytokine Profile

Serum levels of Th1 (TNF-*α* and IFN-*γ*), Th2 (IL-4), Th17 (IL-6 and IL-17A), and Treg (IL-10) cytokines were assessed by flow cytometry (BD FACSCanto II cytometer, BD Cytometric Bead Array Human Th1/Th2/Th17 Kit, USA). The kit included beads coated with capture antibodies and PE-conjugated antibodies specific for IL-2, IL-4, IL-6, IL-10, TNF-*α*, IFN-*γ*, and IL-17A. The detection limits of the assay were 2.6 pg/mL for IL-2, 4.9 pg/mL for IL-4, 2.4 pg/mL for IL-6, 4.5 pg/mL for IL-10, 3.8 pg/mL for TNF-*α*, 3.7 pg/mL for IFN-*γ*, and 18.9 pg/mL for IL-17A. Data processing was performed using the FCAP Array 3.0 software (BD, USA). Serum concentrations of Th17 cytokine IL-21 (Human IL-21 DuoSet ELISA, R & D Systems, USA) and Treg cytokine TGF-*β*1 (Human TGF-beta 1 DuoSet ELISA, R & D Systems, USA) were determined using PowerWave spectrophotometer (BioTec, USA). The detection limits of the assay were 15.6 pg/mL for IL-21 and 31.2 pg/mL for TGF-*β*1. The predominant serum Th cytokine profile was determined by calculating the ratios between serum levels of cytokines associated with Th1 and Th17 cell subpopulations.

### 2.3. Determination of TLR2 Expression on Peripheral Blood Th Cells

The number of circulating blood Th cells expressing TLR2 (CD4^+^CD282^+^) was assessed by flow cytometry using labelled monoclonal antibodies (BD, USA): APC-H7-conjugated anti-СD45 (clone 2D1, mouse IgG1*κ*), PE-Cy7-conjugated anti-CD4 (clone SK3, mouse IgG1*κ*), and Alexa Fluor 488-conjugated anti-CD282 (clone 11G7, mouse IgG1*κ*). The data were analyzed by the FACSDiva software (BD, USA). At least 100,000 events were acquired for each sample. The results were expressed as the percentage of the CD4^+^ lymphocyte population.

### 2.4. Statistical Analysis

Statistical data processing was performed using the Statistica 6.0 software. To check the normality of the distribution for the quantitative parameters, the Kolmogorov–Smirnov test was used. The results of statistical analysis of data with nonnormal distribution were presented as median, upper, and lower quartiles. Statistical significance of the differences was calculated using Mann–Whitney *U* test. The correlation analysis was done using Spearman's correlation coefficient (*r*). The *p*-value of less than 0.05 was considered to be significant. The receiver operating characteristic (ROC) curve was calculated to measure the sensitivity and specificity of the level of TLR2 expression on circulating CD4^+^ cells as a diagnostic and prognostic biomarker for COPD. An area under the ROC curve (AUC) with a 95% confidence interval (CI) was evaluated. A probability of *p* < 0.05 was considered as the threshold of significance.

## 3. Results

### 3.1. TLR2 Expression on Circulating CD4^+^ Cells in COPD of Varying Severity in relation to the Predominant Serum Th Cytokine Profile

In this study, the groups of patients with predominant Th1 and Th17 cytokine profiles have been identified. Due to the fact that the serum levels of the key Th2 cytokine IL-4 in patients with COPD of varying severity were lower or only slightly different from the values of the control group, patients with a Th2 cytokine profile have not been distinguished.

Differences in the changes of TLR2 expression on circulating CD4^+^ cells were found between COPD patients with Th1 and Th17 cytokine profiles during disease progression (Table 2).

In patients with COPD GOLD II and III with a predominance of Th1 immunoregulatory pathway, the relative number of peripheral blood CD4^+^CD282^+^ cells was statistically significantly elevated by 68% (*p* < 0.05) and 92% (*p* < 0.05), respectively, compared to the control group. In patients with COPD GOLD II and III with Th17 cytokine profile, the percentage of the studied cells was statistically significantly increased by 141% (*p* < 0.001) and 258% (*p* < 0.001), respectively, relative to the control values, as well as by 40% (*p* < 0.01) and 87% (*p* < 0.001), respectively, compared to COPD patients with a Th1 cytokine profile. Thus, the greatest enhancement of TLR2 expression on peripheral blood Th cells during COPD progression was found in patients with a Th17 cytokine profile.

### 3.2. Correlations between TLR2 Expression on Circulating CD4^+^ Cells, Serum Th Cytokine Profile, and Clinical Parameters in COPD of Varying Severity

The results of correlation analysis between the percentage of circulating CD4^+^ cells expressing TLR2, serum levels of Th cytokines, and clinical parameters in COPD of varying severity are presented in Table 3.

In COPD GOLD I, strong positive correlations were revealed between the relative number of circulating Th cells expressing TLR2 and serum levels of IL-6 (*r* = 0.71 and *p* < 0.05), IL-17A (*r* = 0.77 and *p* < 0.05), and IL-21 (*r* = 0.79 and *p* < 0.05). The percentage of peripheral blood CD4^+^CD282^+^ cells was negatively correlated with serum concentration of IL-10 (*r* = −0.63 and *p* < 0.05) in this group of patients. In COPD GOLD II, there were positive correlations between the relative number of studied cells and serum levels of IL-21 (*r* = 0.85 and *p* < 0.05), IL-6 (*r* = 0.69 and *p* < 0.05), IL-17A (*r* = 0.67 and *p* < 0.05), and IL-10 (*r* = 0.53 and *p* < 0.05). In COPD GOLD III, strong direct correlations were found between the percentage of peripheral blood CD4^+^CD282^+^ cells and serum content of IL-17A (*r* = 0.96 and *p* < 0.05), IL-21 (*r* = 0.94 and *p* < 0.05), and IL-6 (*r* = 0.87 and *p* < 0.05). Increased number of circulating CD4^+^CD282^+^ cells was negatively correlated with the clinical parameters, such as FEV1/FVC (*r* = −0.87 and *p* < 0.05) and FEV1% predicted (*r* = −0.68 and*p* < 0.05).

Therefore, positive correlations were found between the percentage of circulating CD4^+^CD282^+^ cells and serum levels of Th17 cytokines (IL-6, IL-17A, and IL-21) at all stages of COPD. The serum concentration of IL-10 (Treg cytokine) was negatively correlated with the percentage of studied cells in COPD GOLD I and positively correlated with this parameter in COPD GOLD II. Negative correlations were found between TLR2 expression on Th cells and clinical parameters characterizing bronchial obstruction (FEV1/FVC and FEV1% predicted).

### 3.3. Receiver Operating Characteristic Curve Analysis

The AUC value for the level of TLR2 expression on circulating CD4^+^ cells (percentage of CD4^+^CD282^+^ cells in the peripheral blood) as a diagnostic marker for COPD was 0.952 (95% CI: 0.887–1.017 and *p* < 0.05) with a cut-off of 1.4% (discriminating between COPD patients and healthy subjects) of the cells having a sensitivity of 86.7% and a specificity of 100%. The parameter was also found to be a promising prognostic biomarker of COPD progression with an AUC of 0.984 (95% CI: 0.943–1.025 and *p* < 0.05). The optimal cut-off biomarker value to distinguish patients with COPD GOLD II and GOLD III from patients with COPD GOLD I was 1.7% of the cells with a sensitivity of 95.2% and a specificity of 88.9%. Therefore, the biomarker has high diagnostic and prognostic value in COPD.

## 4. Discussion

Currently, it is generally accepted that COPD is a heterogeneous disease that includes several phenotypes characterized by different clinical manifestations and pathophysiological reactions [3–5, 39]. According to modern concepts, COPD phenotypes can be differentiated based on cytokine profiles associated with the development of a certain type of Th immune response [6–8]. Our previous studies have shown that Th1 and Th17 types of immune response determine the clinical course of COPD. It has been found that inflammation developing according to the Th17 type of immune response is characterized by more pronounced airway obstruction and a higher risk of infectious complications compared to Th1-dependent inflammation [39]. Solleiro-Villavicencio et al. showed that biomass smoke-induced COPD is associated with the Th2 cytokine profile [40]. Additionally, there is evidence that patients with asthma-COPD overlap syndrome also have Th2 immune response [41]. However, our study included smokers and ex-smokers with COPD, and no patient with the predominant Th2 cytokine profile was identified. Accumulating evidence demonstrated that COPD progression is associated with the development of the Th17 immunoregulatory pathway and imbalance between Th17 and Treg cell subpopulations [11–13, 42, 43].

Toll-like receptors are known to be implicated in the regulation of cytokine response in adaptive and innate immune cells. According to the results of numerous studies, TLR2 is one of the most important contributors to the pathogenesis of COPD [22–25]. In COPD, ligands for TLR2 can be PAMPs (lipopeptides, glycolipids, lipoteichoic acid (LTA), peptidoglycan (PGN), etc.) of bacteria colonizing the lower airways (*Streptococcus pneumoniae*, nontypeable *Haemophilus influenzae* (NTHi), *Moraxella catarrhalis*, and *Pseudomonas aeruginosa*) [44], as well as DAMPs releasing as a result of lung tissue exposure to negative environmental factors, such as smoking [29] and air pollution (high-mobility group protein B1 (HMGPB1), heat shock proteins (HSP60, HSP70), *β*-defensin, etc.) [45]. Upon binding to a ligand, TLR2 triggers the myeloid differentiation primary response gene 88 (MyD88) dependent signaling pathway culminating in the activation of nuclear transcription factor nuclear factor kappa-light-chain-enhancer of activated B cells (NF-*κ*B) or activating protein-1 (AP-1). The activation of TLR2 signaling in target cells leads to the synthesis of pro-inflammatory mediators, such as TNF-*α*, IL-6, IL-8, chemokine C-C motif receptor 5/regulated on activation, normal T cell expressed and secreted (CCL5/RANTES), and others [14].

Being accumulated in the lungs during the development of the inflammatory response in COPD, TLR2 ligands can also enter the bloodstream and initiate the TLR2 signaling pathway in circulating immune cells. However, there are few studies regarding TLR2 expression on immune blood cells in COPD. Some research results indicate the inhibition of TLR2-dependent cytokine secretion by leukocytes in the last stages of COPD [46]. There is evidence of a decreased number of circulating monocytes expressing TLR2 and an impaired monocyte-dependent immune response as COPD progresses [46–48]. A number of researchers reported an increase in TLR2-mediated cytokine synthesis by blood neutrophils in COPD patients [21, 49]. There are almost no studies on TLR2 expression on circulating Th cells in COPD.

Current research data suggest that TLR2 significantly contributes to the regulation of differentiation and functioning of Th cells [50, 51]. Experimental studies have shown that the initiation of the TLR2 signaling pathway in T-cell receptor (TCR) activated CD4^+^ cells enhances their proliferation, survival, and chemotaxis and modulates cytokine synthesis and other effector functions [50–52]. However, the role of the TLR2 signaling pathway in the development of Th immune response in COPD needs to be elucidated.

We have analyzed the changes in TLR2 expression on circulating CD4^+^ cells in COPD of varying severity in relation to the predominant serum Th cytokine profile. As a result, a statistically significant increase in the number of peripheral blood CD4^+^CD282^+^ cells has been found in patients with COPD GOLD II and III with both Th1 and Th17 cytokine profiles compared to the control group. At the same time, TLR2 expression was significantly higher in patients with COPD GOLD II and III with the Th17 cytokine profile than in COPD patients with the Th1 cytokine profile. It should be noted that patients with Th17 immune response predominated in the groups of patients with COPD GOLD II and III.

Despite the elevated TLR2 expression on T-helpers in COPD patients with the Th1 cytokine profile, no statistically significant correlations between the relative number of circulating CD4^+^ cells expressing TLR2 and the levels of Th1-associated cytokines have been found. The increased expression of TLR2 on T-helpers in patients with Th1 cytokine profile may be related to the mechanisms of regulation of common T-cell functions (proliferation and survival) during the development of inflammation [50, 51].

Our study has shown strong correlations between the relative content of circulating CD4^+^CD282^+^ cells and serum levels of Th17 cytokines (IL-6, IL-17A, and IL-21) in patients with COPD GOLD I. It has been reported that the stimulation of CD4^+^ cells by TLR2 ligands leads to the activation of key transcription factors of Th17 cells (retinoic-acid-receptor-related orphan nuclear receptor *γt*(ROR*γt*), ROR*α*, RORC) and the production of effector Th17 cytokines (IL-6, IL-17A, IL-17F, IL-22, and IL-21) [33–36, 53]. Consequently, the TLR2/MyD88 signaling pathway is already involved in the development of the Th17 immunoregulatory pathway in the early stage of COPD. In the group of patients with COPD GOLD II, a strong correlation has been found between the percentage of Th cells expressing TLR2 and the concentration of IL-21 promoting Th17 cell differentiation that indicates the involvement of TLR2 in maintaining Th17 immune response during COPD progression. In patients with COPD GOLD III, the relative number of Th cells expressing TLR2 was strongly correlated with levels of IL-6, IL-21, and IL-17A.

Thus, our findings suggest an important contribution of TLR2 to the progression of COPD associated with Th17 immune response. Our data are consistent with accumulated evidence from *in vitro* studies confirming that the activation of TLR2 signaling pathway on CD4^+^ cells promotes the development of Th17 immune response in a number of pathologies, including acute disseminated encephalomyelitis [33], multiple sclerosis [37], systemic lupus erythematosus [36], hypersensitivity pneumonitis [34], and hepatitis B [35, 38].

In our study, correlations between the levels of TLR2 expression on circulating Th cells and the serum content of the anti-inflammatory cytokine IL-10 have been identified. This cytokine is responsible for the limitation of tissue damage during the inflammatory process by inhibiting the macrophage activity and production of TNF-*α*, IL-6, and other pro-inflammatory cytokines [54]. Available data on the systemic levels of IL-10 in COPD are rather contradictory. A number of studies have demonstrated high serum IL-10 levels in patients with COPD [55,56]. At the same time, there are publications indicating a low concentration of IL-10 in the blood in severe COPD [54]. Additionally, there is evidence of the lack of changes in IL-10 levels with the pathology progression [57]. These findings indicate the complex mechanisms of the participation of this cytokine in the pathogenesis of COPD.

Our analysis has shown that the percentage of CD4^+^CD282^+^ cells was inversely correlated with IL-10 levels in the peripheral blood in COPD GOLD I. The serum concentration of this cytokine was positively correlated with the percentage of CD4^+^CD282^+^ cells in COPD GOLD II. It can be assumed that an increase in IL-10 levels serves as a compensatory mechanism aimed at maintaining the balance between pro- and anti-inflammatory mediators during COPD progression. In the group of patients with COPD GOLD III, there was no correlation between the number of CD4^+^CD282^+^ cells and IL-10 levels. It may be due to glucocorticoids used in the therapy of COPD GOLD III. These drugs are known to have potent immunoregulatory effects, so they could influence IL-10 levels in these patients [58].

IL-10 is one of the important cytokines produced by Tregs that play a unique role in controlling the inflammatory response. Currently, there is increasing evidence that TLR2 ligands are involved in the direct modulation of Treg cell functions. However, the data presented in the literature are in marked contrast. In particular, TLR2 stimulation has been shown to reduce the suppressive activity of activated Tregs through mechanisms that are not fully understood yet [59–61]. Nyirenda et al. have shown that the stimulation of TLR2 on Tregs leads to the polarization of these cells into Th17-like phenotype through an increase in the production of IL-6 that exerts an autocrine effect on these cells and promotes switching of the Jak/STAT5/FoxP3 signaling pathway to the Jak/STAT3/RORC pathway [37, 53]. Thus, according to modern concepts, TLR2 may be responsible for a decrease in the suppressive function of Tregs, regulating the Treg/Th17 balance [37, 53, 62]. At the same time, Zanin-Zhorov et al. have shown the opposite effect of Treg activation by endogenous TLR2 ligands that led to an increase in its suppressive action mediated by IL-10 production [52]. In addition, there is evidence that activation of the TLR2 signaling pathway can enhance Treg proliferation and IL-10 synthesis by these cells [63]. The positive and negative effects of TLR2 ligands on Tregs are intriguing and may be associated with using different ligands (synthetic and endogenous) and experimental conditions. Still, further research is needed to understand fully the role of the TLR2 signaling pathway in Treg functioning. It should be noted that in addition to Tregs, other cell subpopulations can make a significant contribution to the production and secretion of IL-10. Therefore, TLR2 signaling appears to be involved in the control of Th17/Treg balance in COPD, but the exact mechanism needs to be further clarified.

Negative correlations were found between TLR2 expression on peripheral blood Th cells and clinical parameters characterizing bronchial obstruction (FEV1/FVC and FEV1% predicted) in patients with COPD. These data indicate an important role of the TLR2 signaling pathway in the development of local inflammation in COPD and confirm our assumption about its participation in the progression of this disease. The results of ROC curve analysis suggest the possibility of using the level of TLR2 expression on peripheral blood T-helpers for diagnosis and predicting the prognosis of COPD.

The strength of this study is a new viewpoint on the role of innate immunity receptors in the development of T-helper immune response in COPD, which will expand the understanding of the pathogenesis of the disease. The limitation is that we have studied the mechanisms of TLR2-mediated regulation of Th immune response in COPD in the peripheral blood, without regard to local inflammation. Another limitation is the inclusion of only smokers and ex-smokers with COPD in the study and the absence of a cohort of nonsmokers with COPD. An additional limitation is the lack of data on the intracellular expression of cytokines in CD4^+^ cells. Further studies devoted to this issue will make it possible to determine more precise mechanisms of the regulation of Th immune response in COPD.

## 5. Conclusion

It can be concluded that increased TLR2 expression on CD4^+^ cells contributes to the polarization of the Th immune response toward the Th17 pathway in COPD, which ultimately leads to the deterioration of respiratory function and the progression of this pathology. However, the exact mechanisms of this process require further research. The level of TLR2 expression on circulating T-helper cells can be used as a diagnostic and prognostic marker in COPD. More in-depth studies will help answer the question of whether TLR2 is a promising therapeutic target for the treatment of this disease.

## Figures and Tables

**Table 1:** Clinical characteristics of the examined groups.

Characteristics	Control group	COPD GOLD I	COPD GOLD II	COPD GOLD III
Total number	97	107	132	84
Sex (male/female)	56/41	63/44	74/58	49/35
Age (years)	51.3 ± 2.5	55.9 ± 2.5	56.8 ± 4.2	61.3 ± 2.4
Smoking status (smokers/ex-smokers)	0	85/22	94/38	59/25
Smoking index (pack/year)	0	24.3 ± 6.5	27.3 ± 5.2	29.4 ± 5.9
Postbronchodilator FEV1 (% of the normal)	104.7 ± 2.0	90.2 ± 3.1	72.6 ± 2.4^*∗*^	41.5 ± 2.8^*∗*^
Postbronchodilator FEV1 (% predicted)	80.75 ± 3.1	61.3 ± 2.4^*∗*^	55.9 ± 1.8^*∗*^	43.7 ± 2.0^*∗*^
mMRC test (score)	0	1.0 ± 0.6	1.45 ± 0.3	1.73 ± 0.8
CAT (score)	0	4.3 ± 1.0	8.5 ± 0.9	12.6 ± 0.1
Number of exacerbations per year	0	0	0.9 ± 0.2	1.9 ± 0.5

*Notes.* Data are presented as mean ± standard deviation. Statistical significance of differences in comparison with the control group (Student's *t*-test): ^*∗*^*p* < 0.05.

**Table 2:** The percentage of CD4^+^CD282^+^ cells in the peripheral blood of healthy individuals and patients with COPD of varying severity with Th1 and Th17 cytokine profiles.

Control group	COPD GOLD I, *n* = 107		COPD GOLD II, *n* = 132		COPD GOLD III, *n* = 84	
	Th1 cytokine profile, *n* = 72	Th17 cytokine profile, *n* = 35	Th1 cytokine profile, *n* = 62	Th17 cytokine profile, *n* = 70	Th1 cytokine profile, *n* = 21	Th17 cytokine profile, *n* = 63
1.1 (0.8–1.3)	1.22 (0.96–1.42)	1.57 (1.11–1.72)	1.85^*∗*^ (1.64–2.06)	2.65^*∗∗∗*##^ (2.48–2.89)	2.11^*∗*^ (1.92–2.27)	3.94^*∗∗∗*###^ (3.41–4.3)

*Notes.* Data are presented as median, upper, and lower quartiles. Statistical significance of the differences between the groups of COPD patients and the control group (Mann–Whitney *U* test): ^*∗*^*p* < 0.05 and ^*∗∗∗*^*p* < 0.001. Statistical significance of the differences between the groups of patients with COPD of the same severity but with different Th cytokine profiles (Mann–Whitney *U* test): ^##^*p* < 0.01 and ^###^*p* < 0.001.

**Table 3:** Correlations between the percentage of CD4^+^CD282^+^ cells in the peripheral blood, serum levels of Th cytokines, and clinical parameters in patients with COPD of varying severity.

Parameters	COPD GOLD I	COPD GOLD II	COPD GOLD III
	CD4^+^CD282^+^ (%)		
IL-6 (pg/mL)	0.71	0.69	0.87
IL-10 (pg/mL)	−0.63	0.53	—
IL-17A (pg/mL)	0.77	0.67	0.96
IL-21 (pg/mL)	0.79	0.85	0.94
Postbronchodilator FEV1 (% predicted)	−0.68		
Postbronchodilator FEV1/FVC (%)	−0.87		

*Notes.* Data are presented as Spearman's correlation coefficient (*r*). Statistical significance (Mann–Whitney *U* test): *p* < 0.05.

## Data Availability

Access to data is restricted due to patient privacy.
